# MiR-1178 Promotes the Proliferation, G1/S Transition, Migration and Invasion of Pancreatic Cancer Cells by Targeting CHIP

**DOI:** 10.1371/journal.pone.0116934

**Published:** 2015-01-30

**Authors:** Zhe Cao, Jianwei Xu, Hua Huang, Peng Shen, Lei You, Li Zhou, Lianfang Zheng, Taiping Zhang, Yupei Zhao

**Affiliations:** 1 Department of General Surgery, Peking Union Medical College Hospital, Chinese Academy of Medical Sciences and Peking Union Medical College, Beijing, China; 2 Department of Nuclear Medicine, Peking Union Medical College Hospital, Chinese Academy of Medical Sciences and Peking Union Medical College, Beijing, China; University of Florida, UNITED STATES

## Abstract

CHIP, a co-chaperone protein that interacts with Hsc/Hsp70, has been shown to be under-expressed in pancreatic cancer cells and has demonstrated a potential tumor suppressor property. Nevertheless, the underlying mechanisms of CHIP regulation in pancreatic cancer cells remain unknown. In this study, we found that miR-1178 decreased the translation of the CHIP protein by targeting the 3′-UTR region. We observed that over-expression of miR-1178 facilitated the proliferation, G1/S transition, migration and invasion of pancreatic cancer cells. Conversely, the inhibition of miR-1178 expression significantly suppressed these phenotypes. Furthermore, CHIP over-expression abrogated miR-1178-induced cell proliferation and invasion. Our data suggest that miR-1178 acts as an oncomiR in pancreatic cancer cells by inhibiting CHIP expression.

## Introduction

Pancreatic cancer is the fourth leading cause of cancer-related deaths in the United States [1]. Existing treatments have little effect on improving survival in patients with pancreatic cancer [2–4]. Exploring the mechanisms of the tumorigenesis, progression and metastasis of pancreatic cancer and identifying new therapeutic targets are urgently needed.

CHIP, a co-chaperone protein that interacts with Hsc/Hsp70 [5], promotes the ubiquitination and degradation of numerous crucial cancer-related proteins, such as NF-κB [6, 7], Met [8] and p53 [9–11]. Previously, we found that CHIP suppressed pancreatic cancer cell proliferation, anchorage-independent growth, migration and invasion by mediating the degradation of EGFR. A low expression level of CHIP was correlated with a worsened prognosis in patients with pancreatic cancer [12]. However, the mechanisms of the regulation of CHIP expression in pancreatic cancer cells remain unknown.

MicroRNAs (miRNAs) are small, endogenous, noncoding RNA molecules with an important role in the post-transcriptional regulation of gene expression [13–16]. Guo J *et al*. [17] found that miR-764–5p inhibits the protein translation of CHIP in mice. However, whether miRNAs regulate CHIP expression in human cancer cells has not been shown previously.

In the current study, we performed an *in silico* screen of miRNAs to identify possible regulators of CHIP expression. We found that miR-1178 targets the 3′-UTR of CHIP mRNA and negatively regulates the translation of CHIP. Furthermore, miR-1178 expression contributes to pancreatic cancer cell proliferation, G1/S transition, migration and invasion. We also found that the effects of miR-1178 are reversible by the over-expression of CHIP. Our findings suggest that miR-1178 expression accelerates pancreatic tumorigenesis by the direct inhibition of CHIP expression.

## Materials and Methods

### Cell lines and reagents

The human pancreatic cancer cell lines BxPC-3, PANC-1, SW1990 and MiaPaCa-2 were gifts from Dr. Freiss H (University of Heidelberg, Heidelberg, Germany) [18, 19]. These cell lines were passaged for less than 6 months after resuscitation. No reauthorization was performed. The cells were cultured in RPMI 1640 or Dulbecco’s modified Eagle’s medium (DMEM) supplemented with 10% FBS (both types of medium were from HyClone, Utah, USA), 100 IU/mL penicillin, and 100 μg/mL streptomycin in a humidified incubator with 5% CO_2_ at 37°C.

### Cell transfection

PANC-1 and BxPC-3 cells were seeded into 6-well plates, cultured overnight, and then transfected with miR-1178 mimics, a miR-1178 inhibitor, or their matched negative controls (all from GenePharma, Shanghai, China). Lipofectamine 2000 (Invitrogen, USA) was used for cell transfection according to the manufacturer’s instructions. Cells were collected for further analyses after an additional 48 hours of incubation.

### RNA isolation and quantitative real-time RT-PCR

Total RNA was extracted from transfected cells using TRIzol reagent (Invitrogen, USA) according to the manufacturer’s instructions. The reverse transcription was conducted by using a reverse transcription kit (Promega, Madison, USA). CHIP mRNA levels were measured by quantitative real-time PCR (qRT-PCR) with the SYBR Green PCR Kit (Takara, Japan). The expression levels of mature miR-1178 were quantified by miR-qRT PCR using the Hairpin-it miRNA qPCR Quantitation Kit (GenePharma, Shanghai, China), which contained a stem-loop-like RT primer and PCR primers specific to the various miRNAs or to the U6 RNA internal control. Analyses were performed on the Applied Biosystems StepOne-Plus Real-Time PCR System (Life Technologies, South San Francisco, USA). Fold changes were calculated using the 2^-ΔΔCT^ method. The reverse primers for GAPDH and CHIP were synthesized by Invitrogen (USA). Primer sequences are shown in S1 Table. The expression of miR-1178 was considered high when the expression level was equal to or above the median of the cohort and low when the level was below the median of the cohort. The qRT-PCR analyses were repeated at least 3 times.

### Western blot analyses

After 48 hours of transfection in 6-well plates, cells were lysed with RIPA buffer (Applygen, Beijing, China). Lysates were denatured with sodium dodecyl sulfate (SDS) sample buffer at 100°C for 5 minutes and separated by SDS polyacrylamide gel electrophoresis (SDS-PAGE), followed by transferring to polyvinylidene difluoride (PVDF) membranes (Millipore, MA, USA). After blocking with 5% non-fat dry milk at room temperature for 1 h, the membranes were incubated overnight at 4°C with primary antibodies. The antibodies are shown in S2 Table. After washing with TBST, the membranes were incubated with secondary antibodies (Applygen, Beijing) at room temperature for 1 hour. Protein bands were visualized with the electrochemiluminescence (ECL) detection system, and the expression levels of the proteins were evaluated using Image-Pro Plus 6.0 software (Media Cybernetics, USA). The western blot analyses were repeated at least 3 times.

### Cell proliferation assay

Cell proliferation was analyzed using a cell count kit (CCK-8) (Dojindo, Japan). PANC-1 (4×10^5^ cells/well) and BxPC-3 (5×10^5^ cells/well) cells were transfected in 6-well plates. After 24 hours, the cells were collected and seeded into 96-well plates (1000 cells/well). Cell proliferation was measured each day for 4 days. At each time point, 10 μL/well CCK-8 reagent was added, and the cells were incubated for 2.5 hours at 37°C. Optical density (OD) was measured at 450 nm and 630 nm by a microplate reader (Wellscan MK3, Thermo Labsystems, Finland). The CCK-8 assay was repeated 3 times with six replicates.

### Cell migration and invasion assay

Cell migration and invasion were evaluated using Transwell migration chambers (8 μm pore size; Corning, USA). The membranes for the invasion assay were coated with a diluted ECM solution (Sigma-Aldrich, Shanghai, China). PANC-1 (2×10^4^ cells/well) or BxPC-3 (4×10^4^ cells/well) cells were seeded in the upper portion of a chamber with serum-free medium after transfection. Medium containing 10% FBS served as a chemoattractant in the lower chamber. After 48 hours of incubation at 37°C, non-invaded cells on the top of the membrane were scraped and removed by cotton swabs, and the invaded cells were washed with PBS, fixed with 90% ethyl alcohol, stained with hematoxylin and then counted using light microscopy. The migration and invasion assay was repeated 3 times with duplicate wells.

### Cell cycle analysis

For the cell cycle assay, cells were harvested at 48 hours after transfection and fixed in 70% ethanol at 4°C overnight. After washing twice with PBS, the cells were incubated with 30 μg/mL propidium iodide (PI), 0.2 mg/mL RNase A, and 0.2% Triton X-100 (all from Sigma-Aldrich, USA) at 37°C for 30 minutes. The cells were then analyzed by flow cytometry (BD Biosciences, USA).

### Dual-luciferase assay

To assess whether CHIP is a direct target of miR-1178, the pMIR-REPORT assay was used. The CHIP 3′-UTR vectors containing the wild-type or mutated seed sequence were constructed by and purchased from GenePharma (Shanghai, China). PANC-1 cells were seeded into 12-well plates (1×10^5^ cells/well) and co-transfected with the wild-type or mutant vector and 50 nM miR-1178 mimics or NC mimics using Lipofectamine 2000. At 24 h after transfection, luciferase activity was measured using the Dual-Luciferase Reporter Assay System (Promega, USA) according to the manufacturer’s protocol.

### Statistical analysis

SPSS v. 13.0 software (SPSS, Inc., Chicago, IL) was used for statistical analyses. Continuous data are presented as the mean ± standard deviation (SD) and were compared by Student’s t test or Fisher’s exact test. *P* < 0.05 was considered statistically significant.

## Results

### CHIP is a direct target of miR-1178

**Fig 1:**
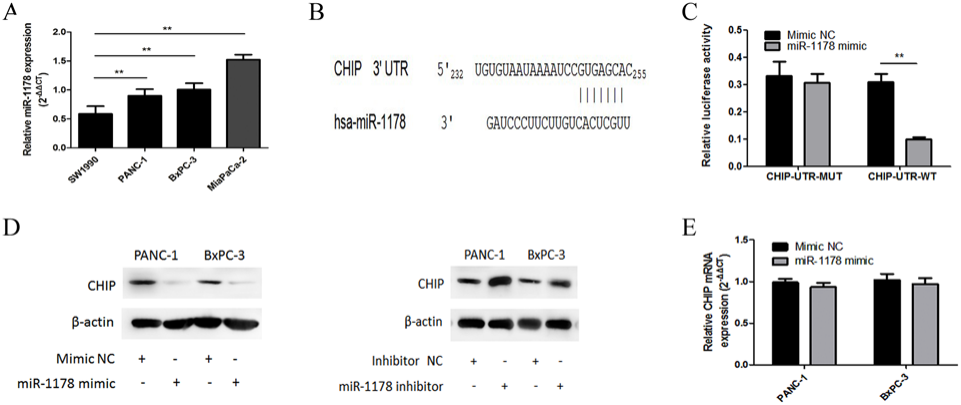
CHIP is a direct target of miR-1178. (A) MiR-1178 expression varied among 4 human pancreatic cancer cell lines. (B) A miR-1178 binding site within the 3′-UTR of CHIP was predicted by TargetScan. (C) In PANC-1 cells, miR-1178 mimics repressed the luciferase activity of the reporter containing the wild-type CHIP 3′-UTR but not the one containing a mutant 3′-UTR. (D) The expression levels of CHIP were detected by western blot after PANC-1 and BxPC-3 cells were transfected with the miR-1178 mimics or the inhibitor. β-actin was used as an internal control. (E) Transfection with the miR-1178 mimics did not suppress the mRNA expression of CHIP. The data are shown as the means ± SD. * *P* ＜ 0.05, ** *P* ＜ 0.01.

To identify regulators of CHIP expression, the open access software TargetScan was used. According to the prediction of TargetScan, miR-1178 was identified as a possible regulator of CHIP expression. We found that miR-1178 expression varied among 4 human pancreatic cancer cell lines, including SW1990, BxPC-3, MiaPaCa-2, and PANC-1, where the relative expression level of miR-1178 was 0.568 ± 0.12, 1.04 ± 0.05, 1.51 ± 0.07, and 0.87 ± 0.09, respectively (Fig. 1A; ***P* < 0.01**). The PANC-1 and BxPC-3 cells, which had moderate expression of miR-1178, were used throughout the rest of the study.

To determine whether CHIP is a direct target of miR-1178, the 3′-UTR of CHIP with wild-type or mutant seed sequence recognition sites was cloned into a dual-luciferase reporter (Fig. 1B). We found that luciferase activity was decreased after co-transfection of the miR-1178 mimics with the wild-type vector, compared with co-transfection of the miR-1178 mimics with the mutant vector (*P* < 0.05) (Fig. 1C). We further evaluated the mRNA and protein levels of CHIP after altering miR-1178 expression. The results showed that over-expression of miR-1178 significantly decreased CHIP protein expression without affecting CHIP mRNA expression, compared with the control. In contrast, down-regulation of miR-1178 increased CHIP protein expression (Fig. 1D, E). These data indicate that miR-1178 can directly target the predicted CHIP seed region.

### MiR-1178 promoted the growth and G1/S transition of PANC1 and BxPC-3 cells

**Fig 2:**
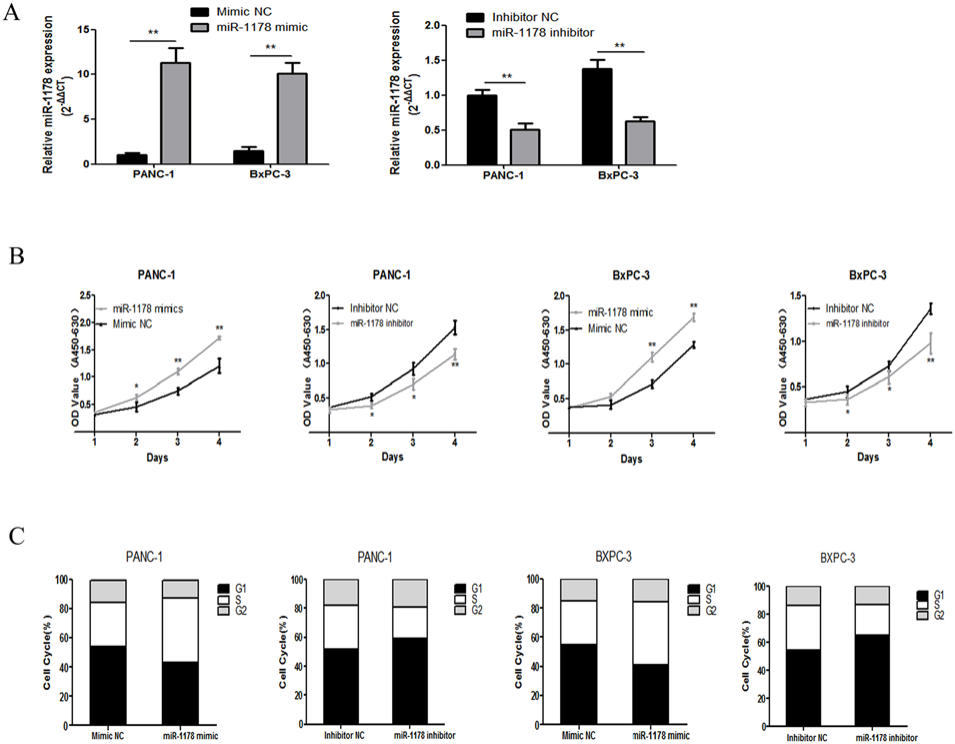
MiR-1178 promotes tumorigenesis in PANC-1 and BxPC-3 cells. (A) qRT-PCR showed that after transfecting cells with the miR-1178 mimics or the inhibitor, the expression of miR-1178 was significantly up-regulated or down-regulated, respectively. (B) A CCK-8 assay showed that miR-1178 over-expression promoted the proliferation of PANC-1 and BxPC-3 cells, while the down-regulation of miR-1178 suppressed cell proliferation. (C) The up-regulation of miR-1178 facilitated the G1/S transition in PANC-1 and BxPC-3 cells. In contrast, a G1/S cell cycle arrest was observed after miR-1178 inhibition. The data are presented as the means ± SD. * *P*＜0. 05, ** *P* < 0. 01.

To explore the function of miR-1178 in pancreatic cancer, miR-1178 mimics and miR-1178 inhibitor were used. After transfection with the miR-1178 mimics or the inhibitor, the expression of miR-1178 was significantly up-regulated or down-regulated, respectively (Fig. 2A). A CCK-8 assay showed that the proliferation rates of PANC-1 and BxPC-3 cells were increased after miR-1178 over-expression. In contrast, miR-1178 down-regulation inhibited the proliferation of pancreatic cancer cells (Fig. 2B). Furthermore, the up-regulation of miR-1178 promoted the G1/S checkpoint transition in both PANC-1 (S phase 30.17 ± 1.31% vs. 39.31 ± 1.51%, *P* < 0.01) and BxPC-3 (S phase 29.89 ± 1.56% vs. 42.57 ± 3.74%, *P* < 0.05) cells, while the down-regulation of miR-1178 led to an elongation of the G1 phase (Fig. 2C).

### MiR-1178 accelerated pancreatic cancer cell migration and invasion

**Fig 3:**
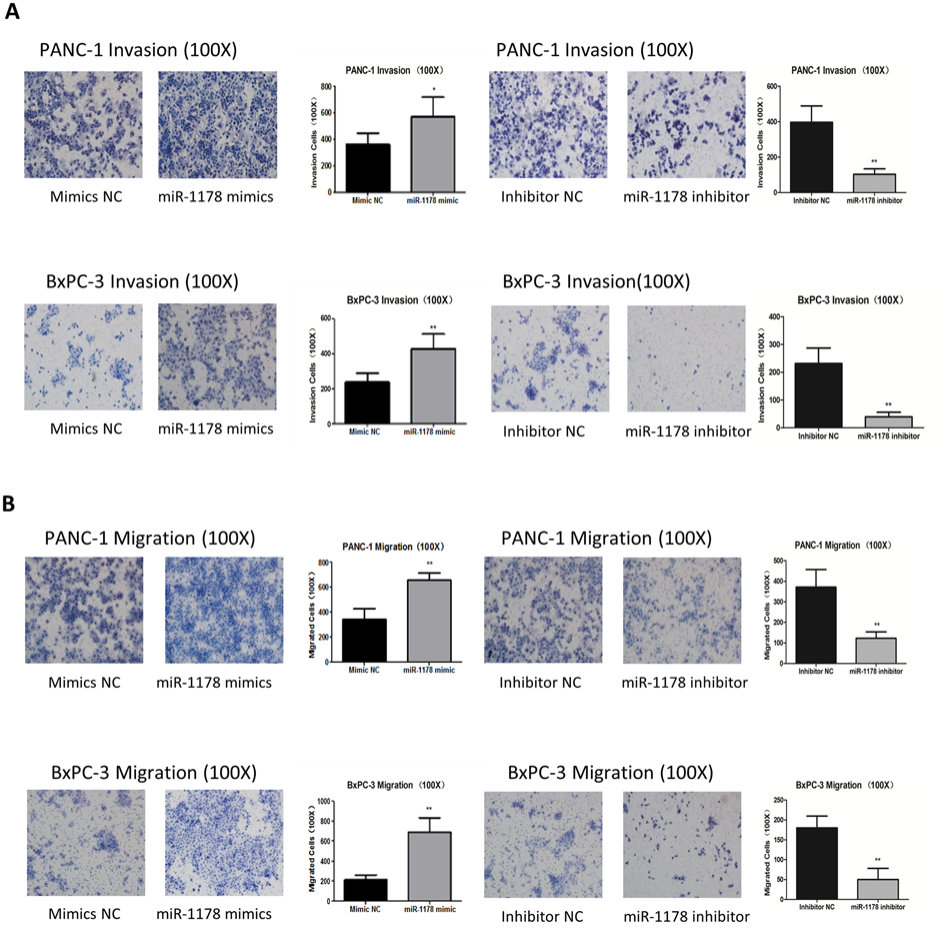
MiR-1178 accelerates pancreatic cancer cell migration and invasion. Cell migration and invasion were analyzed in membrane-containing transwells without or with Matrigel. Cells that had migrated or invaded to the lower surface of the membrane were stained with hematoxylin and eosin and counted under a microscope at 100× magnification. (A) The up-regulation of miR-1178 by transfection of the mimics enhanced cell invasion, while miR-1178 down-regulation deterred cell invasion. (B) Over-expression of miR-1178 promoted cell migration, while the down-regulation of miR-1178 inhibited cell migration. * *P*＜ 0.05, ** *P* < 0.01.

A transwell assay was performed to determine the role of miR-1178 in pancreatic cancer cell migration and invasion. The over-expression of miR-1178 increased the number of PANC-1 cells that penetrated the ECM-coated membrane. In contrast, the invasiveness of PANC-1 cells was significantly decreased when miR-1178 expression was suppressed. Similar results were found in BxPC-3 cells (Fig. 3A). In agreement with this finding, the migratory abilities of these two cell lines were also enhanced after the cells were treated with the miR-1178 mimics, while the opposite results were observed when the cells were treated with the miR-1178 inhibitor (Fig. 3B).

### The downstream signaling pathways of miR-1178

**Fig 4:**
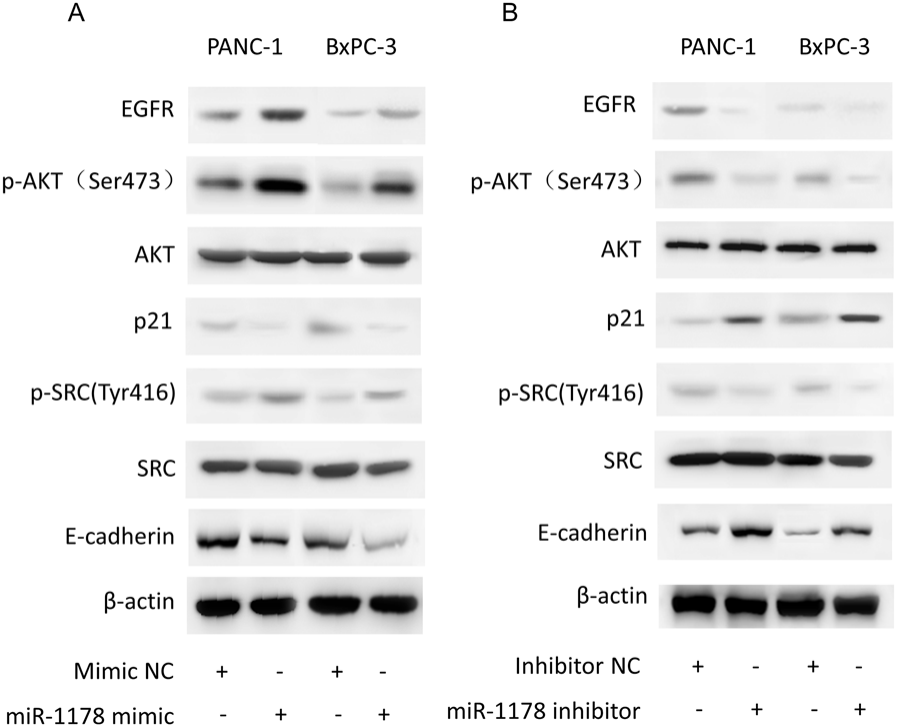
The downstream signaling pathways of miR-1178. (A, B) The expression levels of EGFR, p-AKT, AKT, p21, p-SRC, SRC and E-cadherin were detected by western blot after PANC-1 and BxPC-3 cells were transfected with the miR-1178 mimics or the inhibitor. β-actin was used as an internal control.

In our previous study, we demonstrated that CHIP is a novel tumor suppressor in pancreatic cancer via the degradation of EGFR. Given our observation that miR-1178 down-regulated the expression of CHIP, we hypothesized that miR-1178 might also regulate the activity of EGFR and its downstream signaling cascade. We found that the over-expression of miR-1178 increased the EGFR protein level and activated the downstream AKT/p21 pathway and the Src/E-cadherin pathway (Fig. 4A). In contrast, the inhibition of miR-1178 had the opposite effects (Fig. 4B). Many studies have indicated that the EGFR/AKT/p21 and EGFR/SRC/E-cadherin pathways play key roles in the proliferation, cell cycle control, migration and invasion of pancreatic cancer cells [20–23]. Thus, we speculated that the ectopic activation of the EGFR/AKT/p21 and EGFR/SRC/E-cadherin pathways might partially account for the effects of miR-1178 in pancreatic cancer cells.

### Over-expression of CHIP abrogated miR-1178-induced proliferation, G1/S transition, migration and invasion of pancreatic cancer cells

**Fig 5:**
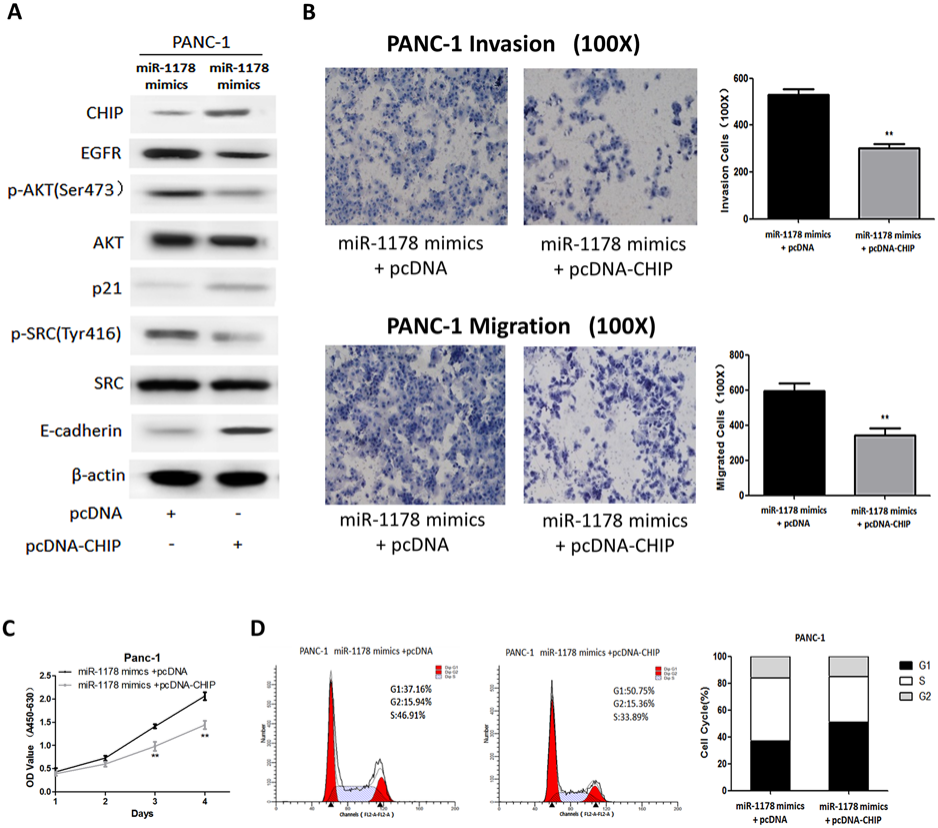
MiR-1178-induced proliferation, G1/S transition, migration and invasion of pancreatic cancer cells is reversible by CHIP over-expression. (A) Western blot analysis of CHIP, EGR, AKT, p21, SRC and E-cadherin expression in PANC-1 cells that were co-transfected with either pcDNA-CHIP or pcDNA empty vector and the miR-1178 mimics. β-actin was also detected as an internal control. (B) Transwell analysis of PANC-1 cells after both co-transfections. (C) Growth curves of PANC-1 cells after co-transfection. (D) The percentage of cells in each phase of the cell cycle was calculated by fluorescence-activated cell sorting (FACS) analysis. * *P* < 0. 05, ** *P* < 0. 01.

To further verify whether miR-1178 promotes a malignant phenotype by repressing CHIP in pancreatic cancer cells, we adopted a “rescue” strategy to examine the functional relevance of the miR-1178/CHIP interaction. The pcDNA-CHIP or the pcDNA empty vector was transfected into PANC-1 cells over-expressing miR-1178. As shown in Fig. 5A, the level of CHIP was increased when pcDNA-CHIP was transfected. Furthermore, the over-expression of CHIP inhibited the miR-1178-induced expression of EGFR, p-AKT and p-SRC. In agreement with the inactivation of EGFR and its downstream pathways, cell invasion, migration (Fig. 5B), proliferation (Fig. 5C) and G1/S transition (Fig. 5D) were all suppressed. These results indicate that miR-1178 promotes cell proliferation, G1/S transition, migration and invasion through the down-regulation of CHIP.

## Discussion

In this study, we showed that miR-1178 targeted the 3′-UTR of the CHIP mRNA, resulting in the inhibition of CHIP protein translation. MiR-1178 facilitated pancreatic cancer cell proliferation, G1/S transition, migration and invasion by repressing CHIP expression. We conclude that miR-1178 is an endogenous attenuator of CHIP expression that promotes a malignant phenotype in pancreatic cancer cells.

The carboxyl terminus of Hsp70-interacting protein (CHIP) is a member of E3 ubiquitin ligase, functioning as a link between the chaperone (heat shock protein 70/90) and proteasome systems. CHIP has been demonstrated to be involved in tumorigenesis, proliferation, and invasion in several malignancies [24], regulating a number of oncogenic proteins including hypoxia-inducible factor 1α (HIF-1α) [25], estrogen receptor-α (ERα) [26], and human telomerase reverse transcriptase [27]. We previously found that CHIP served as a novel tumor suppressor by down-regulating EGFR pathway in pancreatic cancer cells. The expression of CHIP was decreased in pancreatic cancer tissues [13]. However, the mechanism of CHIP low-expression still needs more investigations.

Although increasing evidence has indicated that CHIP plays an important role in cancers [6, 28–30], the mechanisms of CHIP regulation remained poorly understood. Up to now, there were only two published studies focused on the mechanisms of upstream regulation of CHIP. Shimamoto S *et al*. [31] reported that Ca(2+)/S100 bind to the TPR domain and act as upstream regulators of CHIP. Guo J *et al*. [17] showed that miR-764–5p repressed CHIP protein translation in mice through binding to the 3′-UTR of the CHIP mRNA. However, whether miRNAs could regulate CHIP expression in human cancer cells had not been previously determined. Our miRNA screen using TargetScan software predicted that CHIP might be one of the downstream targets of miR-1178. To confirm this prediction, we performed a luciferase reporter assay. We found that luciferase activity was dramatically decreased after co-transfection of the miR-1178 mimics with the vector expressing wild-type CHIP 3′-UTR, compared with co-transfection of the mimics with the vector expressing a mutated CHIP 3′-UTR. Western blot analysis further demonstrated that the over-expression of miR-1178 suppressed CHIP protein expression. In contrast, the down-regulation of miR-1178 increased the CHIP protein level. These results suggest that miR-1178 directly targets the 3′-UTR of CHIP mRNA to inhibit CHIP protein translation. As far as we know, this was the first time that a miRNA which can inhibit the expression of CHIP protein was discovered in human cell lines.

Increasing evidence has shown that miRNAs may have both tumor suppressive [32–34] and oncogenic [35, 36] characteristics in pancreatic cancer cells. However, the studies focuses on the functions of miR-1178 are not reported. In our previous study [12], we found that CHIP suppressed pancreatic cancer cell proliferation, migration and invasion. We therefore hypothesized that miR-1178 might induce pancreatic cancer cell growth, migration and invasion. This hypothesis was supported by our observations. Our data demonstrated that miR-1178 over-expression facilitated pancreatic cancer cell proliferation, G1/S transition, migration and invasion. In contrast, the inhibition of miR-1178 suppressed these processes. Our results suggest that miR-1178 acts as an oncomiR during pancreatic cancer tumorigenesis for the first time.

Pancreatic cancer displays a variety of molecular changes that lead cancer cells not only to survive, but also to invade the surrounding tissues and metastasise to distant sites. The most common alteration involves the epidermal growth factor receptor (EGFR) gene. Li Y *et al*. [37] showed that miR-146a inhibited the invasive capacity of PDAC cells by directly targeting of EGFR. Croce CM [38] reported that miR-21 stimulated EGFR pathway and increased the growth of PDAC cells independent of PTEN degradation. Because our previous study showed that CHIP is a novel post-translational regulator of EGFR [12], we reasoned that miR-1178 might also regulate the activity of EGFR and its downstream signaling cascade. In this study, we showed that miR-1178 over-expression increased the EGFR protein level and activated the downstream AKT/p21 pathway and the Src/E-cadherin pathway. In contrast, the inhibition of miR-1178 had the opposite effects. The activation of AKT/p21 and Src/E-cadherin signaling has been reported to be involved in the proliferation, cell cycle control, migration and invasion of pancreatic cancer cells [20–22, 28]. These results indicate that ectopic activation of the EGFR/AKT/p21 and EGFR/SRC/E-cadherin pathways might partially account for the effects of miR-1178 in pancreatic cancer cells.

To verify whether miR-1178 functions as an oncomiR by repressing CHIP, a “rescue” strategy was adopted to examine the functional relevance of the miR-1178/CHIP interaction. Our results showed that the over-expression of CHIP inhibited the miR-1178-induced expression of EGFR, p-AKT and p-SRC. In accordance with the inactivation of EGFR and its downstream pathways, cell proliferation, G1/S transition, migration and invasion were also decreased. Thus, we concluded that the up-regulated EGFR pathway and facilitation of tumorigenesis by miR-1178 may be due to decreased CHIP expression in pancreatic cancer cells.

In general, *in vitro* data are not sufficient for making clinically relevant inferences in pancreatic cancer [39], especially regarding the correlations between miR-1178 expression and clinicopathological features. In our further study, we will investigate the biological functions of miR-1178 in PDX(patient-derived xenografts) of pancreatic cancer. In addition, we will evaluate the expression of miR-1178 in tissues of pancreatic cancer to investigate the correlation between miR-1178 expression and clinical characteristics of patients with pancreatic cancer.

## Conclusion

In conclusion, the current study demonstrated that miR-1178 promoted pancreatic cancer cell proliferation, G1/S transition, migration and invasion through the down-regulation of CHIP. Our data suggest that miR-1178 is an endogenous attenuator of CHIP that facilitates pancreatic tumorigenesis. To our knowledge, this study is the first to demonstrate the regulation of CHIP expression by miRNA in human cancer cells and clarify the function of miR-1178 in pancreatic cancer. Our results suggest that miR-1178 might serve as a novel therapeutic target for miRNA-based therapy in pancreatic cancer.

## Supporting Information

S1 Table The primer sequences for GAPDH and CHIP. (XLS)

S2 Table The primary antibodies for western blot analysis. (XLS)
